# Identification of Novel Genetic Markers of Breast Cancer Survival

**DOI:** 10.1093/jnci/djv081

**Published:** 2015-04-18

**Authors:** Qi Guo, Marjanka K. Schmidt, Peter Kraft, Sander Canisius, Constance Chen, Sofia Khan, Jonathan Tyrer, Manjeet K. Bolla, Qin Wang, Joe Dennis, Kyriaki Michailidou, Michael Lush, Siddhartha Kar, Jonathan Beesley, Alison M. Dunning, Mitul Shah, Kamila Czene, Hatef Darabi, Mikael Eriksson, Diether Lambrechts, Caroline Weltens, Karin Leunen, Stig E. Bojesen, Børge G. Nordestgaard, Sune F. Nielsen, Henrik Flyger, Jenny Chang-Claude, Anja Rudolph, Petra Seibold, Dieter Flesch-Janys, Carl Blomqvist, Kristiina Aittomäki, Rainer Fagerholm, Taru A. Muranen, Fergus J. Couch, Janet E. Olson, Celine Vachon, Irene L. Andrulis, Julia A. Knight, Gord Glendon, Anna Marie Mulligan, Annegien Broeks, Frans B. Hogervorst, Christopher A. Haiman, Brian E. Henderson, Fredrick Schumacher, Loic Le Marchand, John L. Hopper, Helen Tsimiklis, Carmel Apicella, Melissa C. Southey, Angela Cox, Simon S. Cross, Malcolm W. R. Reed, Graham G. Giles, Roger L. Milne, Catriona McLean, Robert Winqvist, Katri Pylkäs, Arja Jukkola-Vuorinen, Mervi Grip, Maartje J. Hooning, Antoinette Hollestelle, John W. M. Martens, Ans M. W. van den Ouweland, Federik Marme, Andreas Schneeweiss, Rongxi Yang, Barbara Burwinkel, Jonine Figueroa, Stephen J. Chanock, Jolanta Lissowska, Elinor J. Sawyer, Ian Tomlinson, Michael J. Kerin, Nicola Miller, Hermann Brenner, Aida Karina Dieffenbach, Volker Arndt, Bernd Holleczek, Arto Mannermaa, Vesa Kataja, Veli-Matti Kosma, Jaana M. Hartikainen, Jingmei Li, Judith S. Brand, Keith Humphreys, Peter Devilee, Rob A. E. M. Tollenaar, Caroline Seynaeve, Paolo Radice, Paolo Peterlongo, Bernardo Bonanni, Paolo Mariani, Peter A. Fasching, Matthias W. Beckmann, Alexander Hein, Arif B. Ekici, Georgia Chenevix-Trench, Rosemary Balleine, kConFab Investigators, Kelly-Anne Phillips, Javier Benitez, M. Pilar Zamora, Jose Ignacio Arias Perez, Primitiva Menéndez, Anna Jakubowska, Jan Lubinski, Katarzyna Jaworska-Bieniek, Katarzyna Durda, Ute Hamann, Maria Kabisch, Hans Ulrich Ulmer, Thomas Rüdiger, Sara Margolin, Vessela Kristensen, Silje Nord, D. Gareth Evans, Jean E. Abraham, Helena M. Earl, Louise Hiller, Janet A. Dunn, Sarah Bowden, Christine Berg, Daniele Campa, W. Ryan Diver, Susan M. Gapstur, Mia M. Gaudet, Susan E. Hankinson, Robert N. Hoover, Anika Hüsing, Rudolf Kaaks, Mitchell J. Machiela, Walter Willett, Myrto Barrdahl, Federico Canzian, Suet-Feung Chin, Carlos Caldas, David J. Hunter, Sara Lindstrom, Montserrat García-Closas, Per Hall, Douglas F. Easton, Diana M. Eccles, Nazneen Rahman, Heli Nevanlinna, Paul D. P. Pharoah

**Affiliations:** **Affiliations of authors:** Centre for Cancer Genetic Epidemiology, Department of Oncology, University of Cambridge, UK (QG, JT, AMD, MS, JEA, DFE, PDPP); Netherlands Cancer Institute, Antoni van Leeuwenhoek hospital, Amsterdam, the Netherlands (MKS, SC, AB, FBH); Department of Epidemiology, Harvard School of Public Health, Boston, MA (PK, SH, DJH, SL); Program in Genetic Epidemiology and Statistical Genetics, Department of Epidemiology, Harvard School of Public Health, Boston, MA (PK, CCh, DJH, SL); Department of Obstetrics and Gynecology, University of Helsinki and Helsinki University Central Hospital, Helsinki, Finland (SK, RF, TAM, HN); Centre for Cancer Genetic Epidemiology, Department of Public Health and Primary Care, University of Cambridge, Cambridge, UK (MKB, QW, JD, KM, ML, SK, DFE, PDPP); Department of Genetics, QIMR Berghofer Medical Research Institute, Brisbane, Australia (JBee, GCT); Department of Medical Epidemiology and Biostatistics, Karolinska Institutet, Stockholm 17177, Sweden (KC, HD, ME, JiL, JBr, KH, PH); Laboratory for Translational Genetics, Department of Oncology, University of Leuven, Leuven, Belgium (DL); Vesalius Research Center, VIB, Leuven, Belgium (DL); Oncology Department, University Hospital Gasthuisberg, Leuven, Belgium (CW, KL); Copenhagen General Population Study, Herlev Hospital, Copenhagen, Denmark (SEB, BGN, SFN); Department of Clinical Biochemistry, Herlev Hospital, Copenhagen University Hospital, Denmark (SEB, BGN, SFN); Faculty of Health and Medical Sciences, University of Copenhagen, Copenhagen, Denmark (SEB, BGN); Department of Breast Surgery, Herlev Hospital, Copenhagen University Hospital, Denmark (HF); Division of Cancer Epidemiology, German Cancer Research Center (Deutsches Krebsforschungszentrum), Heidelberg, Germany (JCC, AR, PS, DC, AHü, RK, MB); Department of Cancer Epidemiology/Clinical Cancer Registry and Institute for Medical Biometrics and Epidemiology, University Clinic Hamburg-Eppendorf, Hamburg, Germany (DFJ); Department of Oncology, Helsinki University Central Hospital, Helsinki, Finland (CBl); Department of Clinical Genetics, Helsinki University Central Hospital, Helsinki, Finland (KA); Department of Laboratory Medicine and Pathology, Mayo Clinic, Rochester, MN (FJC); Department of Health Sciences Research, Mayo Clinic, Rochester, MN (JEO, CV); Department of Molecular Genetics, University of Toronto, Toronto, Ontario, Canada (ILA); Ontario Cancer Genetics Network, Lunenfeld-Tanenbaum Research Institute of Mount Sinai Hospital, Toronto, Ontario, Canada (ILA, GG); Division of Epidemiology, Dalla Lana School of Public Health, University of Toronto, Toronto, Ontario, Canada (JAK); Prosserman Centre for Health Research, Lunenfeld-Tanenbaum Research Institute, Mount Sinai Hospital, Toronto, Ontario, Canada (JAK); Department of Laboratory Medicine and Pathobiology, University of Toronto, Toronto, Ontario, Canada (AMM); Laboratory Medicine Program, University Health Network, Toronto, Ontario, Canada (AMM); Department of Preventive Medicine, Keck School of Medicine, University of Southern California, Los Angeles, CA (CAH, BEH, FS); University of Hawaii Cancer Centre, Honolulu, HI (LLM); Centre for Epidemiology and Biostatistics, Melbourne School of Population Health, the University of Melbourne, Melbourne, Australia (JLH, CA, GGG, RLM); Genetic Epidemiology Laboratory, Department of Pathology, the University of Melbourne, Melbourne, Australia (HT, MCS); Sheffield Cancer Research Centre, Department of Oncology, University of Sheffield, Sheffield, UK (AC, MWRR); Academic Unit of Pathology, Department of Neuroscience, University of Sheffield, UK (SSC); Cancer Epidemiology Centre, Cancer Council Victoria, Melbourne, Australia (GGG, RLM); Anatomical Pathology, the Alfred Hospital, Melbourne, Australia (CM); Laboratory of Cancer Genetics and Tumor Biology, Department of Clinical Chemistry and Biocenter Oulu, University of Oulu, Oulu, Finland (RW); Laboratory of Cancer Genetics and Tumor Biology, Northern Finland Laboratory Centre NordLab, Oulu, Finland (KP); Department of Oncology, Oulu University Hospital, University of Oulu, Oulu, Finland (AJV); Department of Surgery, Oulu University Hospital, University of Oulu, Oulu, Finland (MG); Department of Medical Oncology, Family Cancer Clinic, Erasmus MC Cancer Institute, Rotterdam, the Netherlands (MJH, AHo, JWMM, AMWvdO); Department of Obstetrics and Gynecology, University of Heidelberg, Heidelberg, Germany (FM, AS, RY, BB); National Center for Tumor Diseases, University of Heidelberg, Heidelberg, Germany (FM, AS); Molecular Epidemiology Group, German Cancer Research Center, Heidelberg, Germany (BB); Division of Cancer Epidemiology and Genetics, National Cancer Institute, Bethesda, MD (JF, SJC); Division of Cancer Epidemiology and Genetics, National Cancer Institute, Rockville, MD (JF); Core Genotyping Facility, Frederick National Laboratory for Cancer Research, Gaithersburg, MD (SJC); Department of Cancer Epidemiology and Prevention, M. Sklodowska-Curie Memorial Cancer Center & Institute of Oncology, Warsaw, Poland (JoL); Division of Cancer Studies, National Institute for Health Research, Comprehensive Biomedical Research Centre, Guy’s & St. Thomas’ NHS Foundation Trust in partnership with King’s College London, London, UK (EJS); Wellcome Trust Centre for Human Genetics and Oxford NIHR Biomedical Research Centre, University of Oxford, UK (IT); Clinical Science Institute, University Hospital Galway, Galway, Ireland (MJK, NM); Division of Clinical Epidemiology and Aging Research, German Cancer Research Center, Heidelberg, Germany (HB, AKD, VA); German Cancer Consortium (DKTK), Heidelberg, Germany (HB, AKD); Saarland Cancer Registry, Saarbrücken, Germany (BH); Imaging Center, Department of Clinical Pathology, Kuopio University Hospital, Kuopio, Finland (AM, VMK, JMH); School of Medicine, Institute of Clinical Medicine, Pathology and Forensic Medicine, University of Eastern Finland, Kuopio, Finland (AM, VMK, JMH); Biocenter Kuopio, Cancer Center of Eastern Finland, Kuopio University Hospital, Kuopio, Finland (VKa); School of Medicine, Institute of Clinical Medicine, Oncology, University of Eastern Finland, Kuopio, Finland (VKa); Department of Human Genetics & Department of Pathology, Leiden University Medical Center, 2300 RC Leiden, the Netherlands (PD); Department of Surgical Oncology, Leiden University Medical Center, 2300 RC Leiden, the Netherlands (RAEMT); Family Cancer Clinic, Department of Medical Oncology, Erasmus MC-Daniel den Hoed Cancer Centrer, Rotterdam, the Netherlands (CS); Unit of Molecular Bases of Genetic Risk and Genetic Testing, Department of Preventive and Predictive Medicine, Fondazione IRCCS Istituto Nazionale dei Tumori, Milan, Italy (PR); IFOM, Fondazione Istituto FIRC di Oncologia Molecolare, Milan, Italy (PP, PM); Division of Cancer Prevention and Genetics, Istituto Europeo di Oncologia, Milan, Italy (BB); Cogentech Cancer Genetic Test Laboratory, Milan, Italy (PM); David Geffen School of Medicine, Department of Medicine, Division of Hematology and Oncology, University of California at Los Angeles, CA (PAF); Department of Gynecology and Obstetrics, University Hospital Erlangen, Friedrich-Alexander University Erlangen-Nuremberg, Comprehensive Cancer Center Erlangen-EMN, Erlangen, Germany (PAF, MWB, AHe); Institute of Human Genetics; University Hospital Erlangen, Friedrich-Alexander University Erlangen-Nuremberg, Comprehensive Cancer Center Erlangen-EMN, Erlangen, Germany (ABE); Western Sydney and Nepean Blue Mountains Local Health Districts, Westmead Millennium Institute for Medical Research, University of Sydney, Sydney, Australia (RB); Peter MacCallum Cancer Center, Melbourne, Australia (kConFab Investigators); the University of Melbourne, Melbourne, Australia (KAP); Division of Cancer Medicine, Peter MacCallum Cancer Centre, Melbourne, Australia (KAP); Centro de Investigación en Red de Enfermedades Raras, Valencia, Spain (JBen); Human Genetics Group, Human Cancer Genetics Program, Spanish National Cancer Research Centre, Madrid, Spain (JBen); Servicio de Oncología Médica, Hospital Universitario La Paz, Madrid, Spain (MPZ); Servicio de Cirugía General y Especialidades, Hospital Monte Naranco, Oviedo, Spain (JIAP); Servicio de Anatomía Patológica, Hospital Monte Naranco, Oviedo, Spain (PM); Department of Genetics and Pathology, Pomeranian Medical University, Szczecin, Poland (AJ, JL, KJB, KD); Molecular Genetics of Breast Cancer, German Cancer Research Center, Heidelberg, Germany (UH, MK); Frauenklinik der Stadtklinik Baden-Baden, Baden-Baden, Germany (HUU); Institute of Pathology, Städtisches Klinikum Karlsruhe, Karlsruhe, Germany (TR); Department of Oncology - Pathology, Karolinska Institutet, Stockholm, Sweden (SM); Department of Genetics, Institute for Cancer Research, Oslo University Hospital, Radiumhospitalet, Oslo, Norway (VKr, SN); Faculty of Medicine (Faculty Division Ahus), University of Oslo, Norway (VKr, SN); Genomic Medicine, Manchester Academic Health Science Centre, University of Manchester, Central Manchester Foundation Trust, St. Mary’s Hospital, Manchester, UK (DGE); Cambridge Breast Research Unit and NIHR Cambridge Biomedical Research Centre, University of Cambridge, Department of Oncology, Cambridge, UK (JEA, HME, CCa); Cambridge Experimental Cancer Medicine Centre, Cambridge, UK (JEA, HME, CCa); Warwick Clinical Trials Unit, University of Warwick, UK (LH, JAD); Cancer Research UK Clinical Trials Unit, Institute for Cancer Studies, the University of Birmingham, Edgbaston, Birmingham, UK (SB); Early Detection Research Group, Division of Cancer Prevention National Cancer Institute Bethesda, MD (CBe); Department of Biology, University of Pisa, Pisa, Italy (DC); Epidemiology Research Program, American Cancer Society, Atlanta, GA (WRD, SMG, MMG); Channing Division of Network Medicine, Department of Medicine, Brigham and Women’s Hospital, Boston, MA (SH); Division of Biostatistics and Epidemiology, University of Massachusetts-Amherst School of Public Health and Health Sciences, Amherst, MA (SH); Division of Cancer Epidemiology and Genetics, National Cancer Institute, Bethesda, MD (RNH, MJM); Department of Nutrition, Harvard School of Public Health, Boston, MA (WW); Genomic Epidemiology Group, German Cancer Research Center, Heidelberg, Germany (FC); Breast Cancer Functional Genomics Laboratory, Cancer Research UK Cambridge Institute, University of Cambridge, Li Ka Shing Centre, UK (SFC, CCa); Breakthrough Breast Cancer Research Centre, Division of Breast Cancer Research, the Institute of Cancer Research, London, UK (MGC); Division of Genetics and Epidemiology, Institute of Cancer Research, Sutton, Surrey, UK (MGC, NR); Faculty of Medicine, University of Southampton, UK (DME).

## Abstract

**Background:**

Survival after a diagnosis of breast cancer varies considerably between patients, and some of this variation may be because of germline genetic variation. We aimed to identify genetic markers associated with breast cancer–specific survival.

**Methods:**

We conducted a large meta-analysis of studies in populations of European ancestry, including 37954 patients with 2900 deaths from breast cancer. Each study had been genotyped for between 200000 and 900000 single nucleotide polymorphisms (SNPs) across the genome; genotypes for nine million common variants were imputed using a common reference panel from the 1000 Genomes Project. We also carried out subtype-specific analyses based on 6881 estrogen receptor (ER)–negative patients (920 events) and 23059 ER-positive patients (1333 events). All statistical tests were two-sided.

**Results:**

We identified one new locus (rs2059614 at 11q24.2) associated with survival in ER-negative breast cancer cases (hazard ratio [HR] = 1.95, 95% confidence interval [CI] = 1.55 to 2.47, *P* = 1.91 x 10^–8^). Genotyping a subset of 2113 case patients, of which 300 were ER negative, provided supporting evidence for the quality of the imputation. The association in this set of case patients was stronger for the observed genotypes than for the imputed genotypes. A second locus (rs148760487 at 2q24.2) was associated at genome-wide statistical significance in initial analyses; the association was similar in ER-positive and ER-negative case patients. Here the results of genotyping suggested that the finding was less robust.

**Conclusions:**

This is currently the largest study investigating genetic variation associated with breast cancer survival. Our results have potential clinical implications, as they confirm that germline genotype can provide prognostic information in addition to standard tumor prognostic factors.

Survival after a diagnosis of breast cancer varies considerably between patients. Many factors influence outcome in an individual patient, including inherited genetic variation. This hypothesis is supported by several lines of evidence. It has been shown that first-degree relatives with breast cancer have a correlated likelihood of dying from the disease (1–3). Additionally, mouse strain is a determinant of metastatic progression in in vivo models (4). There are many mechanisms through which germline genetic variation might affect prognosis. Some known disease susceptibility alleles confer differential risks of different tumor subtypes that are associated with different outcomes—for example, deleterious alleles of *BRCA1* are associated with estrogen receptor (ER)–negative disease, and several common germline genetic variants that are associated with susceptibility to breast cancer have different risks of ER-positive and ER-negative disease (5,6). Germline genotype could also affect the efficacy of adjuvant drug therapies or might influence tumor-host interactions, such as those involving the stroma surrounding a tumor or the host’s immune response (7). The host genotype might also influence the propensity of a tumor to seed and grow at metastatic sites.

The association between common germline genetic variation and breast cancer–specific survival has been examined in many candidate gene studies (8–16). These studies have identified numerous single nucleotide polymorphisms (SNPs) possibly associated with outcome, but none have been conclusively replicated in further studies. Genome-wide association studies (GWAS) have been very successful at identifying susceptibility alleles for a wide range of normal and disease phenotypes (17). However, GWAS of breast cancer survival published to date have had modest sample sizes and have not identified any confirmed associations (7,18). It is clear that the success of other GWAS has depended on large sample sizes. It is likely that large studies of survival time are required if alleles associated with prognosis in breast cancer are to be identified. We therefore pooled genotype data from multiple breast cancer GWAS discovery and replication efforts and linked these data to available survival time data for the case patients in order to maximize statistical power to detect associations.

## Methods

### Breast Cancer Patient Samples

We pooled data from multiple breast cancer case cohorts in populations of European ancestry with existing high-density SNP genotyping (Supplementary Table 1–4, available online). These data comprise eight main genotype datasets (Collaborative Oncological Gene-environment Study [COGS] (5), BPC3 (19), CGEMS (20), HElsinki Breast Cancer Study [HEBCS] (16), METABRIC (21), PG-SNPs (22–25), Sweden Breast Cancer Study [SASBAC] (26), and UK2 (27)). Each study had been genotyped for 200000 to 900000 SNPs across the genome using a variety of genotyping arrays. SASBAC and HEBCS are single-case cohorts, and all others have multiple constituent studies. A summary of the studies in COGS contributing data to our analysis is shown in Supplementary Table 3 (available online). ER status was obtained mostly from medical records followed by immunohistochemistry performed on tumor tissue microarrays or whole-section tumor slides. All studies were approved by the relevant institutional review boards, and all participants provided written informed consent.

### Genotyping Quality Control

The genotype and the sample quality control (QC) have been previously described for COGS (5), CGEMS (20), HEBCS (16), SASBAC (26), UK2 (27), PG-SNPs (22), and BPC3 (19). QC procedures have not been described previously for the Molecular Taxonomy of Breast Cancer International Consortium (METABRIC) germline genotype data: SNPs were excluded 1) if the genotype frequencies deviated from those expected under Hardy-Weinberg equilibrium at *P* values of less than 1×10^−5^, 2) if they had a minor allele frequencies (MAFs) of less than 1%, 3) if the MAF was between 1% and 5% and call rate was under 99%, and 4) if the MAF was greater than 5% and the call rate was under 95%. A summary of the number of genotyped SNPs and the number of SNPs passing QC is shown in Supplementary Table 4 (available online). All individuals with low call rates (< 95%) or high or low heterozygosity (*P* < 1 x 10^−5^) were excluded from subsequent analyses. All analyses were based on subjects of European ancestry based on genotype data. The methods and criteria for exclusion of non-European samples has been described previously for all studies apart from METABRIC, for which we used a set of unlinked SNPs, and the program Local Ancestry in adMixed Populations (28) to assign intercontinental ancestry based on the HapMap release no.22 genotype frequency data for European, African, and Asian populations. Subjects with less than 90% European ancestry were excluded.

### Imputation

Genotypes for common variants across the genome were imputed using a reference panel from the 1000 Genomes Project in order to increase genome coverage. Genotype imputation for PG-SNPs, METABRIC, UK2, SASBAC, HEBCS, and COGS was performed using IMPUTE2 (29) after prephasing with SHAPEIT (30). This was done in chunks of 5 MB and default parameters for both programs. The imputation reference set consisted of 2184 phased haplotypes from the full 1000 Genomes Project data set (March 2012). All genomic locations are given in NCBI Build 37/UCSC hg19 coordinates. Imputation for CGEMS and BPC3 was performed using the program MaCH (31). SNPs with imputation *r* ^2^ < 0.3 were excluded on a study-by-study basis. All SNPS with a MAF of less than 1% were excluded.

### Statistical Analysis

The primary end point was breast cancer–specific survival. Time-to-event was calculated from the date of diagnosis. However, case patients were recruited at variable times before or after diagnosis; therefore, time under observation was calculated from date of recruitment (left censoring) in order to prevent the bias that could result from the inclusion of prevalent cases. Follow-up was right censored on the date of death if death was from something other than breast cancer, the date last known alive if death did not occur, or at 10 years after diagnosis, whichever came first. We fitted univariate Cox proportional hazard models to assess the association of genotype with breast cancer–specific mortality. We also ran analyses for ER-negative and ER-positive breast cancer. Each data set including the three component case cohorts in BPC3 was analyzed separately. The Cox models were stratified by study for the COGS dataset. We controlled for cryptic population substructure by including a variable number of principal components as covariates for each data set. The Cox proportional hazards assumption was tested for each significant SNP of interest analytically using Schoenfeld residuals. There was no evidence of nonproportional hazards. For the statistically significantly associated SNPs, we ran multivariable Cox models adjusting for age, nodal status, tumor size, tumor grade, and adjuvant treatment using the COGS data. We used an in-house program written in C++ for the analysis of COGS, HEBCS, METABRIC, PG-SNPs, SASBAC, and UK2. Analysis of CGEMS and BPC3 data was conducted using ProbABEL (32). We excluded SNPs with MAFs under 1% because of extreme value of the test statistics. Overall statistical significance tests for each SNP were performed by combining the results for each data set using a fixed-effects meta-analysis. All statistical tests were two-sided. Inflation of the test statistics (λ) was estimated by dividing the 45th percentile of the test statistic by 0.357 (the 45th percentile for a χ2 distribution on 1 degree of freedom). Heterogeneity between studies was measured using the *I* ^2^ statistic (33,34). Correlation between SNPs was calculated using Pearson correlation coefficient. Associations were regarded as statistically significant at a nominal *P* value of less than 5 x 10^–8^ (genome-wide significance).

### eQTL

Expression quantitative trait locus (eQTL) analyses were performed for all genes in the 1 MB region spanning the associated SNPs using probe-level gene expression data for breast epithelium samples taken from normal tissue adjacent to the tumor of 135 breast cancer patients of European ancestry from the METABRIC study (21). These were assayed using the Illumina HT12 platform. We also analyzed eQTL data of 387 breast tumors from the Cancer Genome Atlas (TCGA) (303 ER-positive, 81 ER-negative, three unknown) assayed using the Agilent G4502A-07-3 array (35). Germline SNP genotypes were available for normal and tumor samples from the Affymetrix SNP 6.0 platform imputed into 1000 Genomes Project data (March 2012) for the three SNPs of interest: rs2059614 at 11q24.2, and rs148760487 and rs114860916 at 2q24.2 (see Results section). Association between genotype and expression was tested by linear regression with false discovery rate control.

## Results

The overall results were based on 37954 case patients with 2900 deaths from breast cancer (Supplementary Table 2, available online). The results of the subtype-specific analyses were based on 23059 ER-positive case patients (1333 deaths) from five studies and 6881 ER-negative case patients (920 deaths) from eight studies.

In the overall analysis, we identified 28 SNPs associated with breast cancer–specific survival at *P* values of less than 5x10^-8^ (Table 1; Supplementary Figures 1 and 2, available online). All 28 SNPs were located in the same region on chromosome 2 and had been imputed in all eight datasets. The strongest association was for rs148760487 (hazard ratio [HR] = 1.88, 95% confidence interval [CI] = 1.51 to 2.34), *P* = 1.5x10^-8^) (risk allele frequency = 0.01). This SNP was associated with breast cancer–specific survival in both ER-positive (HR = 2.07, 95% CI = 1.47 to 2.91, *P* = 3.1x10^-5^) and ER-negative case patients (HR = 1.87, 95% CI = 1.27 to 2.75, *P* = .002). The imputation efficiency for these SNPs varied between an *r* ^2^ of 0.69 and 0.997 for the eight data sets. The inflation factor λ for the overall analysis was 1.01.

**Table 1.** Summary of SNPs by levels of statistical significance in the final combined analysis, ER-negative and ER-positive case patients*

*P* value cutoff	All invasive case patients		ER-positive case patients		ER-negative case patients	
	Observed	Expected	Observed	Expected	Observed	Expected
< 5x10^-8^	28	0	0	0	1	0
< 1x10^-7^	31	0	0	0	3	1
< 1x10^-6^	34	10	2	10	25	10
< 1x10^-5^	134	97	75	98	292	100
< 1x10^-4^	928	971	894	975	1164	1002
< 1x10^-3^	9375	9707	10 509	9752	11 032	10 020

* ER = estrogen receptor; SNP = single nucleotide polymorphism.

**Figure 1.**
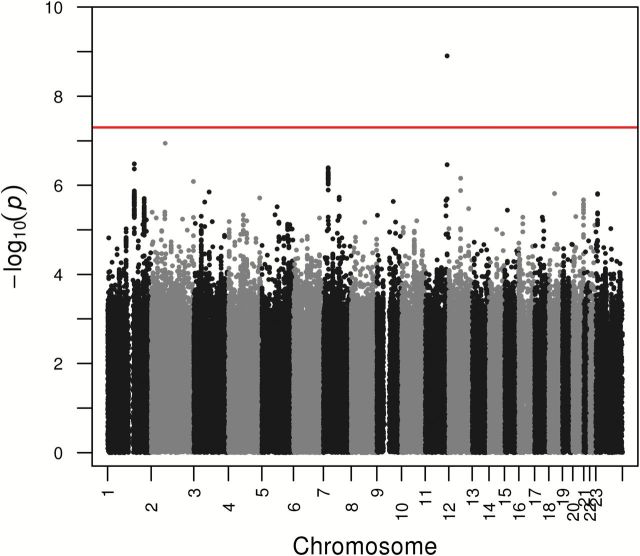
Association plot for combined GWAS and COGS analyses for estrogen receptor (ER)–negative cases. The *P* values of the association between each single nucleotide polymorphism (SNP) and breast cancer survival were obtained by cox regression analyses with adjustment for principle components for each study and then combined. The y-axis shows the -log_10_ *P* values of each SNP analyzed, and the x-axis shows their chromosome position. The **red horizontal line** represents *P* = 5x10^-8^. All statistical tests were two-sided.

**Figure 2.**
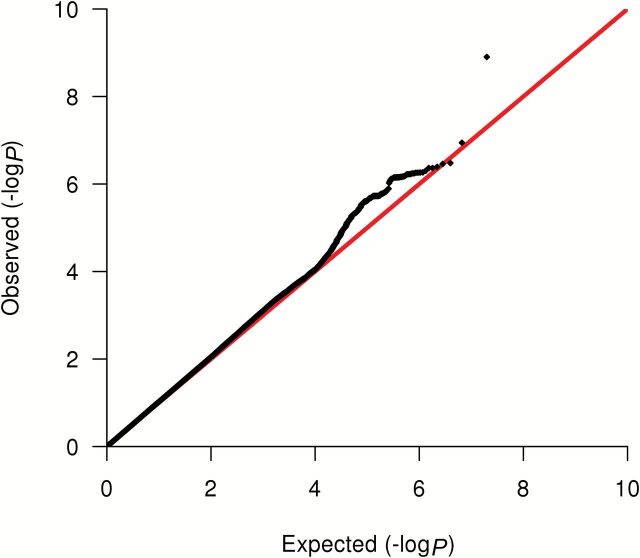
Quantile-Quantile (Q-Q) plot for the combined GWAS and COGS analyses for estrogen receptor (ER)–negative cases. The y-axis represents the observed -log_10_ *P* value, and the x-axis represents the expected -log_10_ *P* value. The **red line** represents the expected distribution under the null hypothesis of no association. All statistical tests were two-sided.

A single imputed SNP, rs2059614, located on chromosome 11, was associated with breast cancer–specific mortality at genome-wide statistical significance in patients with ER-negative disease (HR = 1.90, 95% CI = 1.54 to 2.33, *P* = 1.3x10^-9^) (risk allele frequency = 0.06) (Table 1; Figures 1 and 2). The imputation *r* ^2^ ranged from 0.75 to 0.82 across eight studies with ER-negative cases. The inflation factor λ for analysis based on ER-negative cases was 1.03. No SNP reached nominal genome-wide statistical significance in the analysis of case patients with ER-positive disease (Supplementary Figures 3 and 4, available online), for which the strongest association was for rs7149859 in chromosome 14 (HR = 1.22, 95% CI = 1.13 to 1.33, *P* = 7.0x10^-7^). There was very little between study heterogeneity for the overall analysis for rs148760487 (I^2^ = 0%, *P* = .59) (Supplementary Figure 5, available online) or the ER-negative analysis for rs2059614 (I^2^ = 0%, *P* = .50) (Supplementary Figure 6, available online).

We conducted follow-up imputation on the two regions around rs148760487 and rs2059614 using the IMPUTE2 Markov chain Monte Carlo algorithm with 80 iterations without prephasing, as omitting the prephasing step should maximize imputation accuracy (29). We reimputed all SNPs in the genomic regions 500 KB pairs on either side of the two SNPs of interest. The association for rs148760487 was somewhat weaker (HR = 1.75, 95% CI = 1.39 to 2.20, *P* = 1.44 x 10^–6^). A highly correlated SNP, rs114860916 (*r* ^*2*^ = 0.97) was now the most strongly associated SNP in the region (HR = 1.74, 95% CI = 1.39 to 2.18, *P* = 1.16 x 10^–6^). In contrast, rs2059614 remained the most strongly associated SNP with survival of ER-negative disease (HR = 1.95, 95% CI = 1.55 to 2.47, *P* = 1.91 x 10^–8^). Again there was no evidence of heterogeneity in the meta-analysis of these SNPs (data not shown).

We genotyped rs148760487 and rs2059614 in 2113 breast cancer case patients from the Studies of Epidemiology and Risk Factors in Cancer Heredity (SEARCH) in order to confirm the quality of the imputation. The correlation between the imputed and observed genotypes was 0.63 for rs148760487 and 0.68 for rs2059614. This compares with an estimated imputation *r* ^2^ of 0.76 and 0.79 for the genotypes imputed with prephasing using genotype data from the COGS custom array. We then compared the results of association analyses for the SEARCH data set using the imputed and observed genotypes. For rs148760487, there were 133 breast cancer deaths. In this subset the association based on genotyped data was weaker than the association based on the imputed data (HR = 1.66, 95% CI = 0.75 to 3.69, *P* = .21 and HR = 2.06, 95% CI = 0.84 to 5.04, *P* = .11, respectively), but this difference was not statistically significant (*P* = .72). For rs2059614, there were genotyped and imputed data for 300 ER-negative samples with 45 deaths. The association with genotyped data was stronger than that for imputed data (HR = 1.80, 95% CI = 0.99 to 3.25, *P* = .05 and HR = 1.44, 95% CI = 0.51 to 4.12, *P* = .49, respectively), as would be expected for a true positive association. Again, this difference was not statistically significant (*P* = .72). We also conducted multivariable analysis for these two SNPs using the pooled data within the COGS dataset, stratified by study and adjusting for principal components, age, lymph node status, tumor size, stage, grade, ER status (where applicable), and adjuvant treatment; the results were similar to the main findings (data not shown). Finally, we compared the hazard ratios for rs148760487 in all case patients and rs2059614 for ER-negative case patients in premenopausal (defined as age at diagnosis younger than 45 years) and postmenopausal (age at diagnosis of 55 years or older). There was no statistically significant difference (*P* = .96 and .24, respectively).

The risk allele of rs2059614 was associated with increased expression of *EI24* and *CHEK1* in normal breast epithelium adjacent to tumor from the METABRIC study (*P* = .002 and .007, respectively) (Supplementary Figure 7, available online). *EI24* is a tumor suppressor gene involved in *TP53* dependent apoptosis. *CHEK1* is required for checkpoint-mediated cell cycle arrest in response to DNA damage. Other SNPs in the region were more strongly associated with both *EI24* and *CHEK1* expression, but were not associated with prognosis. There were no statistically significant eQTLs for rs148760487 and rs114860916 in normal breast epithelium. None of the three SNPs had statistically significant eQTLs in tumors from the TCGA study. We also explored the association between gene expression for all genes in the 1 MB region spanning the associated SNPs and breast cancer–specific mortality using KM plotter (36). Data were available for 575 ER-negative breast cancer patients. *CHEK1* expression was not associated with relapse-free survival in ER-negative case patients (HR = 0.86, 95% CI = 0.65 to 1.12, *P* = .25) (Supplementary Figure 8, available online) but had statistically significant associations in ER-positive case patients (HR = 1.59, 95% CI = 1.31 to 1.91, *P* = 1.2x10^-6^). *EI24* expression was associated with relapse-free survival in both ER-positive case patients (HR = 0.75, 95% CI = 0.63 to 0.90, *P* = .002) and ER-negative case patients (HR = 1.38, 95% CI = 1.07 to 1.77, *P* = .01). It is interesting that the direction of association is consistent: The risk allele G of rs2059614 is associated both with poor breast cancer–specific survival in ER-negative case patients and with higher levels of *EI2*4 expression in normal breast epithelium, which in turn is associated with poorer relapse-free survival in breast cancer. Expression of neither of the genes near rs148760487 was associated with relapse.

Both the two top SNPs lie in putative enhancer sequences for which promoter interactions have been predicted (Supplementary Figure 9, available online) (37,38). *IFIH* or *FAP* might be the target of rs148760487, and *EI24* might be the target of rs2059614 because the SNP is in an enhancer in endothelial cells that is predicted to regulate *EI24*.

## Discussion

This is the largest genetic association study of breast cancer prognosis to date. We identified one new locus (rs148760487 at 2q24.2) associated with breast cancer–specific survival in all breast cancer and one new locus (rs2059614 at 11q24.2) associated with breast cancer survival in ER-negative case patients at genome-wide levels of statistical significance. However, both these associations were based on imputed genotype data. Genotyping a subset of the case patients confirmed that the quality of the imputation was reasonable, but for one SNP (rs148760487), the association in the subset of samples with both genotyped and imputed data was weaker for the genotyped data. Thus we are less confident that this represents a true positive. On the other hand, as would be expected for a true positive, the association of rs2059614 got stronger when comparing genotyped with imputed data, suggesting that this is a robust association.

Two genes lie within the 1 MB region on chromosome 2 spanning rs148760487 to *KCNH7* and *BC042876. KCNH7* encodes a voltage-gated potassium channel with diverse functions and has no obvious role in cancer. *BC042876* is a noncoding RNA gene with no known function. Another SNP in the same region, rs1424760, has been reported to be associated with serum phospholipid levels, but this SNP is only weakly correlated with rs148760487 (*r* ^2^ = 0.11).

There are 18 genes in the genomic region 500 KB either side of rs2059614 in chromosome 11 (Supplementary Table 5, available online). Several of these are known to be involved in processes relevant to cancer, such as cell death and DNA damage responses. Of particular interest are *EI24* and *CHEK1,* as expression of both of these in normal breast epithelium is associated with rs2059614 genotype. Additionally, expression of *EI24* is associated with relapse-free survival in both ER-positive case patients and ER-negative case patients. Furthermore, this genomic region, 11q24, is frequently altered in cancers.

Genome-wide association studies with large-scale replication have been extremely successful in identifying multiple variants associated with many different phenotypes. For example, more than 70 common variants are known to be associated with an altered risk of breast cancer (5,6). In contrast, this study of breast cancer prognosis has identified just two variants associated at genome-wide statistical significance. There are several possible reasons for this difference. Despite the large sample size used in these analyses, the power to detect association with breast cancer–specific survival is only modest (see Supplementary Figure 10, available online). All of the common alleles associated with disease susceptibility confer relative risks of less than 1.2, and most are associated with relative risks of less than 1.1. Alleles such as these can be detected using case-control studies with a total sample size of approximately 100000 (5). However, our analyses, based on 2900 breast cancer deaths, had limited power to detect alleles conferring hazard ratios of less than 1.2. Power to detect an allele with a hazard ratio greater than 1.5 was good (60% power if the MAF = 0.05, 100% power if the MAF > 0.1), suggesting that few such alleles are likely to exist.

Another issue affecting our ability to detect associations with prognosis is the heterogeneity of the phenotype. A wide variety of factors influence survival time after diagnosis, including tumor biology and treatment. Breast cancer is a heterogeneous disease, and different disease subtypes have different clinical outcomes (39–41). Restricting the analyses to specific subtypes in addition to ER status would reduce this heterogeneity, but the sample size would also be greatly reduced as subtype-specific information is not available for all case patients in these analyses, and some subtypes are relatively uncommon.

Our findings provide support for the hypothesis that germ line genetic variation influences outcome after a diagnosis of breast cancer. Identification of novel germline genetic markers of breast cancer prognosis may help to elucidate molecular mechanisms of tumor progression and metastasis. Ultimately this may lead to the identification of new targets for therapeutic interventions. It may also lead to insights into mechanisms driving the differential response to adjuvant therapies and thereby enable improved targeting of therapy. In the clinical setting, germline markers of prognosis could be used to enhance risk stratification and provide patients with information about their prognosis in order to identify those patients most likely to benefit from adjuvant therapy. However, even studies larger than ours will be required in order to meet the challenge of identifying additional loci. Genotyping samples from clinical trials may prove to be particularly useful, but it is clear that data from multiple studies will need to be combined if there are to be further successes in this field.

## Funding

Higher-level funding: The COGS project was funded through a European Commission’s Seventh Framework Programme grant (agreement number 223175 - HEALTH-F2-2009–223175). The Breast Cancer Association Consortium (BCAC) is funded by Cancer Research-UK (C1287/A10118 and C1287/A12014). Meetings of the BCAC have been funded by the European Union COST programme (BM0606). ELAN Program of the University Hospital Erlangen (BBCC).

Personal support: DFE is a Principal Research Fellow of Cancer Research UK. JLH is a National Health and Medical Research Council (NHMRC) Australia Fellow. MCS is an NHMRC Senior Research Fellow. GCT is an NHMRC Senior Principal Research Fellow. DL is supported by the FWO and the KULPFV/10/016-SymBioSysII. JL is a UNESCO-L’Oréal International Fellow. RB was a Cancer Institute NSW Fellow. KAP is a National Breast Cancer Foundation Fellow (Australia).

Funding of constituent studies (these are listed by funding agency, with each grant number in parentheses): Academy of Finland (266528); Addenbrookes Charitable Trust; Agency for Science, Technology and Research of Singapore; Asociación Española Contra el Cáncer and the Fondo de Investigación Sanitario (PI11/00923, PI08/1120); Baden Württemberg Ministry of Science, Research and Arts; Breast Cancer Campaign (2009PR42); Breast Cancer Research Foundation; Canadian Institutes of Health Research (CIHR Team in Familial Risks of Breast Cancer program); Cancer Australia; Cancer Councils of New South Wales, Victoria, Tasmania and South Australia; Cancer Foundation of Western Australia; Cancer Fund of North Savo; Cancer Research UK (C1287/A10118, C1287/A12014, A7572, A10124, A11699, A16561, C507/A6306, C10097/A7484,C1275/A11699); Chief Physician Johan Boserup and Lise Boserup Fund; Danish Breast Cancer Group; Danish Medical Research Council; Deutsche Krebshilfe (70-2892-BR I, PBZ_KBN_122/P05/2004); Dietmar-Hopp Foundation; Dutch Cancer Society (1997-1505, 2004–3124, NKI2007-3839, 2009–4318, NKI2009-4363); Dutch government (NWO 184.021.007); Dutch National Genomics Initiative; ELAN-Fond of the University Hospital of Erlangen; European Community′s Seventh Framework Programme (HEALTH-F2-2009–223175); Federal Ministry of Education and Research, Germany (01KH0402); Finnish Cancer Society; Fondazione IRCCS Istituto Nazionale Tumori; Genome Spain Foundation; German Cancer Research Center (DKFZ); Hamburg Cancer Society; Helmholtz Society; Helsinki University Central Hospital Research Fund; Italian Association for Cancer Research (AIRC); Kuopio University Hospital special Government Funding; National Health and Medical Research Council of Australia (209057, 251553 and 504711); National Breast Cancer Foundation (Australia); National Institute for Health Research, Cambridge Biomedical Research Centre; Nordic Cancer Union; Märit and Hans Rausings Initiative Against Breast Cancer; Nordic Cancer Union; Polish Foundation of Science (PBZ_KBN_122/P05/2004); Queensland Cancer Fund; Red Temática de Investigación Cooperativa en Cáncer; Sigrid Juselius Foundation; Susan G. Komen Breast Cancer Foundation; Stichting tegen Kanker (232–2008 and 196–2010); United States National Institutes of Health (BBMRI-NL-CP16, CA69638, CA69417, CA06503, CA116201, CA122340, CA128978, CA63464, CA54281, CA098758, CA132839, CA164920, CA98216, CA098233, CA148065, CA98710, CA98758, and the Intramural Research Program of the National Institutes of Health and the National Cancer Institute); UK National Institute for Health Research Biomedical Research Centres at the University of Cambridge, Guy’s & St. Thomas’ NHS Foundation Trust in partnership with King’s College London, and University of Oxford; University of Eastern Finland strategic funding; Victorian Health Promotion Foundation; Victorian Breast Cancer Research Consortium; Yorkshire Cancer Research (S295, S299, S305PA).

## Supplementary Material

Supplementary Data

## Notes

The study sponsors had no role in the design of the study; the collection, analysis, or interpretation of the data; the writing of the manuscript; nor the decision to submit the manuscript for publication. For full names of abbreviated study names see Supplementary Material.

This study would not have been possible without the contributions of the following: DC Tessier, F. Bacot, D. Vincent, S. LaBoissière, and F. Robidoux and the staff of the genotyping unit, (Genome Quebec); J. Stone, S. McBean, J. Hadlington, A. Mustafa, and K. Cook (Illumina); M. Angelakos, J. Maskiell, G. Dite, T. Selander, N. Weerasooriya (ABCFS); S. Cornelissen, R. van Hien, L. Braaf, F. Hogervorst, S. Verhoef, E. Rutgers, F. Atsma (ABCS); N. McInerney, G. Colleran, A. Rowan, A. Jones (BIGGS); P. Bugert (BSUCH); DU Andersen, MB Arnadottir, A. Bank, DK Hansen (CGPS); G. Pita, C. Alonso, D. Herrero, N. Álvarez, P. Zamora, P. Menendez (CNIO-BCS); H. Ziegler, S. Wolf, V. Hermann (ESTHER); D. Greco, K. von Smitten, I. Erkkilä, T. Heikkinen, K. Aaltonen (HEBCS); E. Myöhänen, H. Kemiläinen (KBCP); G. Peuteman, D. Smeets, T. Van Brussel, K. Corthouts (LMBC); J. Heinz, N. Obi, A. Vrieling, S. Behrens, U. Eilber, M. Celik, T. Olchers (MARIE); S. Manoukian, B. Peissel, G. Scuvera, D. Zaffaroni, M. Barile, I. Feroce, L. Bernard (MBCSG); K. Mononen, M. Otsukka (OBCS); T. Selander, N. Weerasooriya (OFBCR); E. Krol-Warmerdam, J. Blom. J. Molenaar (ORIGO); L. Brinton, M. Sherman, N. Szeszenia-Dabrowska, B. Peplonska, W. Zatonski, P. Chao, M. Stagner (PBCS); P. Bos, J. Blom, E. Crepin, A. Nieuwlaat, A. Heemskerk (RBCS); S. Higham, I. Brock, S. Balasubramanian, D. Connley, H. Cramp (SBCS); J. Young, C. Twelves, AL Vallier, S. Ingle, R. Hardy (PGSNPS); M. Rübner, S. Landrith, S. Oeser, L. Häberle (BBCC).
