# Supplementary material for: Identification of Novel Genetic Markers of Breast Cancer Survival
Source: J Natl Cancer Inst. 2015 Apr 18;107(5):djv081. doi: 10.1093/jnci/djv081 (PMC4555642; doi:10.1093/jnci/djv081)
Supplement: Supplementary Data [file supp_107_5_djv081__index.html]

Identification of Novel Genetic Markers of Breast Cancer Survival — Supplementary Data 

# Identification of Novel Genetic Markers of Breast Cancer Survival

## Supplementary Data

Data files

**Files in this Data Supplement:**

- Supplementary Data - Supplementary Data
