# Supplementary material for: Identification of Novel Genetic Markers of Breast Cancer Survival
Source: J Natl Cancer Inst. 2015 Apr 18;107(5):djv081. doi: 10.1093/jnci/djv081 (PMC4555642; doi:10.1093/jnci/djv081)
Supplement: Supplementary Data [file supp_djv081_14_0511R1_Schmidt_supp_mat_final.doc]

**Supplementary Data**

**Guo and Schmidt et al. Identification of novel genetic markers of breast cancer survival**

**List of Tables**

**Supplementary Table 1.** Summary of the participating studies

**Supplementary Table 2.** Summary of samples included in the GWAS and COGS contributing data for analysis

**Supplementary Table 3.** Summary of studies in COGS contributing to overall and ER-negative breast cancer-specific survival study

**Supplementary Table 4.** Summary of the number of genotyped SNPs and the number of SNPs passing QC for each study

**Supplementary Table 5.** List of genes found within genomic region 500KB either side of rs148760487 in chromosome 2 and rs2059614 in chromosome 11

**List of Figures**

**Supplementary Figure 1.** Association plot for the final combined GWAS and COGS analyses for all breast cancer cases

**Supplementary Figure 2.** Q-Q plot for the combined GWAS and COGS analyses for all breast cancer cases

**Supplementary Figure 3.** Association plot for the final combined GWAS and COGS analyses for ER- positive cases

**Supplementary Figure 4.** Q-Q plot for the combined GWAS and COGS analyses for ER-positive cases

**Supplementary Figure 5.** Per-allele breast cancer-specific survival HR for rs148760487 on chromosome 2 by study based on imputed data with pre-phasing

**Supplementary Figure 6.** Per-allele breast cancer-specific survival HR for rs2059614 on chromosome 11 by study for ER-negative breast cancer patients, based on imputed data with pre-phasing

**Supplementary Figure 7.** Box plot of A) *EI24* and B) *CHEK1* expression by rs2059614 genotype in normal breast epithelium from the METABRIC study

**Supplementary Figure 8.** Kaplan-Meier plot of effect of *CHEK1* and *EI24* on relapse-free survival of breast cancer patients using KM Plotter^34^: A) effect of *CHEK1* in ER-positive patients; B) effect of *CHEK1* in ER-negative patients; C) effect of *EI24* in ER-positive patients; D) effect of *EI24* in ER-negative patients

**Supplementary Figure 9.** Regional association plots displays the strength of genetic association (-log10 P) versus chromosomal position (MB), where each dot represents an imputed SNP. The purple horizontal line represents the threshold for genome-wide significance (P = 5 × 10^-8^). Gene structures are depicted with the direction of transcription indicated by arrows. Enhancer locations, as defined in references,^41,42^ are shown where elements overlapping the best associated SNPs are labelled with their predicted target genes, *IFIH1* and *FAP* in (A). In (B), the blue stripe marks the position of rs2059614, and the red and green enhancers are predicted to target *EI24* and *CHEK1*, respectively. Positions of poorly imputed, common SNPs are shown.

**Supplementary Figure 10**. Power plots for A) all breast cancers cases; B) ER-negative breast cancer cases

**Supplementary table 1.** Summary of the participating studies

3

| **Study** | **Genotyping platform** | **Age** | **Country** | **Description of the study** |
| --- | --- | --- | --- | --- |
| COGS | Illumina iSelect | 16-96 | International | Collaborative Oncological Gene-Environment Study^5^ |
| CGEMS | Illumina Hap550K | 44-83 | USA | NHS cases from Nurses Health Study genotyped as part of CGEMS project^20^ |
| SASBAC | Illumina HumanHap300 and HumanHap240S | 50-75 | Sweden | Cases from Swedish Case-control study, part of BCAC^26^ |
| UK2 | Illumina 670k | 17-88 | UK | Consist of National study of breast cancer of age < 41 years, and Subset of samples from national familial breast cancer study^27^ |
| Metabric | Affymetrix SNP 6.0 | 26-96 | UK | UK samples from international breast cancer genomics project^21^ |
| PG-SNPs | Affymetrix SNP 6.0 | 22-77 | UK | UK samples from breast cancer chemotherapy treatment response study^22-25^ |
| HEBCS | Illumina 550K | 26-87 | Finland | Helsinki Breast Cancer Study^16, 38-40^ |
| BPC3-CPSII | Illumina 660W | 51-89 | USA | The National Cancer Institute Breast and Prostate Cancer Cohort Consortium: American Cancer Society Cancer Prevention Study-II^19^ |
| BPC3-EPIC | Illumina 660W | 27-75 | Europe | The National Cancer Institute Breast and Prostate CancerCohort Consortium: European Prospective Investigation of Cancer^19^ |
| BPC3-NHS2 | Illumina 550K | 44-83 | USA | The National Cancer Institute Breast and Prostate CancerCohort Consortium: Nurses' Health Studies II^19^ |
|  |  |  |  |  |

**Supplementary Table 2.** Summary of samples included in the GWAS and COGS contributing data for analysis

| **Study** | **All cases** |  | **ER positive** |  | **ER negative** |  |
| --- | --- | --- | --- | --- | --- | --- |
|  | **N (breast cancer deaths)** | **Person-years** | **N (breast cancer deaths)** | **Person-years** | **N (breast cancer deaths)** | **Person-years** |
| COGS | 29,360 (1790) | 128,552 | 20,605 (942) | 89,702 | 4,926 (558) | 21,437 |
| CGEMS | 1,145 (93) | 7,711 | -- | -- | -- | -- |
| SASBAC | 787 (69) | 3,739 | 483 (44) | 2,294 | 108 (9) | 502 |
| UK2 | 2,763 (233) | 23,112 | -- | -- |  |  |
| Metabric | 369 (86) | 1,570 | 291 (59) | 1,268 | 63 (25) | 225 |
| PG-SNPs | 1,786 (204) | 5,820 | 1,188 (116) | 3,916 | 586 (87) | 1,888 |
| HEBCS | 742 (285) | 4,666 | 492 (172) | 3,458 | 196 (101) | 982 |
| BPC3-CPSII | 293 (30) | 2,544 | -- | -- | 293 (30) | 2,544 |
| BPC3-EPIC | 476 (74) | 2,226 | -- | -- | 476 (74) | 2,226 |
| BPC3-NHS2 | 233 (36) | 2,732 | -- | -- | 233 (36) | 2,732 |
| **Total** | **37,954 (2,900)** |  | **23,059 (1,333)** |  | **6,881 (920)** |  |

ER=estrogen receptor

**Supplementary Table 3.** Summary of studies in COGS contributing to overall and ER-negative breast cancer-specific survival study

| **Study Acronym** | **Study** | **Country** | **Study design** |
| --- | --- | --- | --- |
| ABCFS | Australian Breast Cancer Family Study | Australia | Cancer registries in Victoria and New South Wales (1992-1999): all cases from Melbourne and Sydney diagnosed before age 40 plus a random sample of those diagnosed at ages 40-59. |
| ABCS | Amsterdam Breast Cancer Study | Netherlands | Breast cancer patients diagnosed before age 50 in 2003-2009 at the NKI-AVL; and (ABCS-F) All non-BRCA1/2 breast cancer cases from the family cancer clinic of the NKI-AVL tested in the period 1995-2009; all ages and diagnosed with breast cancer in 1965-2008. |
| BBCC | Bavarian Breast Cancer Cases and Controls | Germany | Consecutive, unselected cases with invasive breast cancer recruited at the University Breast Centre, Franconia in Northern Bavaria from 2002-2010. |
| BIGGS | Breast Cancer in Galway Genetic Study | Ireland | Unselected cases recruited from University College Hospital Galway and surrounding hospitals in the West of Ireland since 2001. |
| CGPS | Copenhagen General Population Study | Denmark | Consecutive, incident cases from one hospital with centralized care for a population of 400,000 women in Copenhagen (2001-present). |
| ESTHER | ESTHER Breast Cancer Study | Germany | Breast cancer cases in all hospitals in the state of Saarland, from 2001-2003 (ESTHER) and 1996-1998 (VERDI) |
| HEBCS | Helsinki Breast Cancer Study | Finland | (1) Consecutive cases (883) from the Department of Oncology, Helsinki University Central Hospital 1997-8 and 2000, (2) Consecutive cases (986) from the Department of Surgery, Helsinki University Central Hospital 2001–2004, (3) Familial breast cancer patients (536) from the Helsinki University Central Hospital, Departments of Oncology and Clinical Genetics (1995-). |
| KARBAC | Karolinska Breast Cancer Study | Sweden | 1. Familial cases from Department of Clinical Genetics, Karolinska University Hospital, Stockholm. 2. Consecutive cases from Department of Oncology, Huddinge & Söder Hospital, Stockholm 1998-2000. |
| KBCP | Kuopio Breast Cancer Project | Finland | Women seen at Kuopio University Hospital between 1990-1995 because of a breast lump, mammographic abnormality, or other breast symptom and who were found to have breast Cancer. |
| kConFab/AOCS | Kathleen Cuningham Foundation Consortium for Research into Familial Breast Cancer / Australian Ovarian Cancer Study | Australia | Index (youngest affected) cases from BRCA1-and BRCA2-mutation-negative multiple-case breast and breast-ovarian families recruited though family cancer clinics from across Australia and New Zealand from 1998-present. |
| LMBC | Leuven Multidisciplinary Breast Centre | Belgium | All patients diagnosed with breast cancer and seen in the Multidisciplinary Breast Centre in Leuven (Gashuisberg) since June 2007 plus retrospective collection of cases diagnosed since 2000. |
| MARIE | Mammary Carcinoma Risk Factor Investigation | Germany | Incident cases diagnosed from 2001-2005 in the study region Hamburg in Northern Germany, and from 2002-2005 in the study region Rhein-Neckar-Karlsruhe in Southern Germany. |
| MCCS | Melbourne Collaborative Cohort Study | Australia | Incident cases from the cohort of 24,469 women, diagnosed during the follow-up from baseline (1990-1994) to 2008. |
| MEC | Multiethnic Cohort | USA | Incident cases identified from SEER cancer registries in Los Angeles County & State registries in California & Hawaii, USA from 1993-2002. Grouped by self-reported ethnicity. |
| OBCS | Oulu Breast Cancer Study | Finland | Consecutive incident cases diagnosed at the Oulu University Hospital between 2000-2004. |
| OFBCR | Ontario Familial Breast Cancer Registry | Canada | Invasive cases aged 20-54 years identified from the Ontario Cancer Registry from 1996-1998. All those at high genetic risk were eligible; random samples of women not meeting these criteria were also asked to participate. |
| ORIGO | Leiden University Medical Centre Breast Cancer Study | Netherlands | Consecutive case patients diagnosed 1996–2006 in 2 hospitals in South–West Netherlands (Leiden & Rotterdam). No selection for family history; Rotterdam case patients selected for diagnosis aged < 70 years. Case patients with in situ carcinomas eligible. |
| pKARMA | Karolinska Mammography Project for Risk Prediction of Breast Cancer-prevalent case | Sweden | Incident cases from Jan 2001–Dec 2008 from the Stockholm/Gotland area. Identified through the Stockholm breast cancer registry. |
| RBCS | Rotterdam Breast Cancer Study | Netherlands | Familial breast cancer patients selected from the clinical genetics centre at Erasmus Medical Centre between 1994-2005. |
| SASBAC | Singapore and Sweden Breast Cancer Study | Sweden | Women diagnosed in Sweden aged 50-74 in 1993-1995. |
| SEARCH | Study of Epidemiology & Risk Factors in Cancer Heredity | UK | Identified through the Eastern Cancer Registration and Information Centre: (I) prevalent Cases; diagnosed 1991-1996; under 55 years of age at diagnosis; recruited 1996-2002 (ii) incidence cases; diagnosed since 1996; under 70 years of age at diagnosis; recruited 1996-present. |
| SKKDKFZS | Städtisches Klinikum Karlsruhe Deutsches Krebsforschungszentrum Study | Germany | Women diagnosed with primary in situ or invasive breast cancer at the Städtisches Klinikum Karlsruhe from March 1993 to July 2005. Cases were 21-93 years of age. |

**Supplementary Table 4.** Summary of the number of genotyped SNPs and the number of SNPs passing QC for each study

| **Study** | **Number of SNPs genotyped** | **Number of genotyped SNPs**  **passing QC** |
| --- | --- | --- |
| COGS | 211,155 | 199,961 |
| CGEMS | 555,352 | 546,646 |
| SASBAC | 574,017 | 522,095 |
| UK2 | 536,243 | 531,424 |
| Metabric | 906,115 | 715,952 |
| PG-SNPs | 906,115 | 695,738 |
| HEBCS | 302,597 | 301,530 |
| BPC3-CPSII | 557,466 | 534,154 |
| BPC3-EPIC | 560,763 | 534,927 |
| BPC3-NHS2 | 519,021 | 501,085 |

**Supplementary Table 5.** List of genes found within genomic region 500KB either side of rs148760487 in chromosome 2 and rs2059614 in chromosome 11

| **Gene** | **Official full name** | **Location** | **Gene type** | **Description** |
| --- | --- | --- | --- | --- |
| *KCNH7* | potassium voltage-gated channel, subfamily H (eag-related), member 7 | 2q24.2 | protein coding | Voltage-gated potassium (Kv) channels represent the most complex class of voltage-gated ion channels from both functional and structural standpoints. Their diverse functions include regulating neurotransmitter release, heart rate, insulin secretion, neuronal excitability, epithelial electrolyte transport, smooth muscle contraction, and cell volume. This gene encodes a member of the potassium channel, voltage-gated, subfamily H. This member is a pore-forming (alpha) subunit. There are at least two alternatively spliced transcript variants derived from this gene and encoding distinct isoforms. |
| *BC042876* | uncharacterized LOC101929570 | 2q24.2 | ncRNA | Homo sapiens cDNA clone |
| *ROBO4* | roundabout, axon guidance receptor, homolog 4 (Drosophila) | 11q24.2 | Protein coding |  |
| *HEPN1* | hepatocellular carcinoma, down-regulated 1 | 11q24 | protein coding | This gene is expressed in the liver, and encodes a short peptide that is localized predominantly to the cytoplasm. Transient transfection studies showed that expression of this gene significantly inhibited cell growth, and it may have a role in apoptosis. Expression of this gene is downregulated or lost in hepatocellular carcinomas (HCC), suggesting that loss of this gene is involved in carcinogenesis of hepatocytes. Also to note is that this gene maps to the 3'-noncoding region of HEPACAM gene on the antisense strand. |
| *HEPACAM* | hepatic and glial cell adhesion molecule | 11q24.2 | protein coding | he protein encoded by this gene is a single-pass type I membrane protein that localizes to the cytoplasmic side of the cell membrane. The encoded protein acts as a homodimer and is involved in cell motility and cell-matrix interactions. The expression of this gene is downregulated or undetectable in many cancer cell lines, so this may be a tumor suppressor gene |
| *CCDC15* | coiled-coil domain containing 15 | 11q24.2 | protein coding |  |
| 1. *SLC37A2* | - - 1. solute carrier family 37 (glucose-6-phosphate transporter) | 1. 11q24.2 | 1. protein coding |  |
| *TMEM218* | transmembrane protein 218 | 1. 11q24.2 | 1. protein coding |  |
|  |  |  |  |  |

| *PKNOX2* | PBX/knotted 1 homeobox 2 | 1. 11q24.2 | 1. protein coding | Homeodomain proteins are sequence-specific transcription factors that share a highly conserved DNA-binding domain and play fundamental roles in cell proliferation, differentiation, and death. PKNOX2 belongs to the TALE (3-amino acid loop extension) class of homeodomain proteins characterized by a 3-amino acid extension between alpha helices 1 and 2 within the homeodomain. |
| --- | --- | --- | --- | --- |
| *FEZ1* | fasciculation and elongation protein zeta 1 (zygin I) | 1. 11q24.2 | 1. protein coding | This gene is an ortholog of the C. elegans unc-76 gene, which is necessary for normal axonal bundling and elongation within axon bundles. Expression of this gene in C. elegans unc-76 mutants can restore to the mutants partial locomotion and axonal fasciculation, suggesting that it also functions in axonal outgrowth. The N-terminal half of the gene product is highly acidic. Alternatively spliced transcript variants encoding different isoforms of this protein have been described. |
| *MGC39545* | uncharacterized LOC403312 | 1. 11q24.2 | unknown |  |
| *EI24* | etoposide induced 2.4 | 1. 11q24 | 1. protein coding | This gene encodes a putative tumor suppressor and has higher expression in p53-expressing cells than in control cells and is an immediate-early induction target of p53-mediated apoptosis. The encoded protein may suppress cell growth by inducing apoptotic cell death through the caspase 9 and mitochondrial pathways. This gene is located on human chromosome 11q24, a region frequently altered in cancers. Alternative splicing results in multiple transcript variants. Pseudogenes of this gene have been defined on chromosomes 1, 3, 7, and 8. |
| *STT3A* | subunit of the oligosaccharyltransferase complex (catalytic) | 1. 11q23.3 | 1. protein coding |  |
| *CHEK1* | checkpoint kinase 1 | 11q24.2 | protein coding | The protein encoded by this gene belongs to the Ser/Thr protein kinase family. It is required for checkpoint mediated cell cycle arrest in response to DNA damage or the presence of unreplicated DNA. This protein acts to integrate signals from ATM and ATR, two cell cycle proteins involved in DNA damage responses, that also associate with chromatin in meiotic prophase I. Phosphorylation of CDC25A protein phosphatase by this protein is required for cells to delay cell cycle progression in response to double-strand DNA breaks. Several alternatively spliced transcript variants have been found for this gene. |
|  |  |  |  |  |

| *ACRV1* | acrosomal vesicle protein 1 | 11q24.2 | protein coding | This gene encodes a testis-specific, differentiation antigen, acrosomal vesicle protein 1, that arises within the acrosomal vesicle during spermatogenesis, and is associated with the acrosomal membranes and matrix of mature sperm. The acrosomal vesicle protein 1 may be involved in sperm-zona binding or penetration. Alternatively spliced transcript variants have been described. |
| --- | --- | --- | --- | --- |
| *PATE1* | prostate and testis expressed 1 | 11q24.2 | protein coding |  |
| *PATE2* | prostate and testis expressed 2 | 11q24.2 | protein coding |  |
| *PATE3* | prostate and testis expressed 3 | 11q24.2 | protein coding |  |
| *PATE4* | prostate and testis expressed 4 | 11q24.2 | protein coding |  |
| *HYLS1* | hydrolethalus syndrome 1 | 11q24.2 | protein coding | This gene encodes a protein localized to the cytoplasm. Mutations in this gene are associated with hydrolethalus syndrome. Multiple alternatively spliced variants, encoding the same protein, have been identified. |
|  |  |  |  |  |

**Supplementary Figure 1.** Association plot for the final combined GWAS and COGS analyses for all breast cancer cases. The P values of the association between each SNP and breast cancer survival were obtained by cox regression analyses with adjustment for principle components for each study and then combined. The *y* axis shows the –log_10_P values of each SNP analyzed, and the *x* axis shows their chromosome position. The red horizontal line represents P=5x10^-8^.


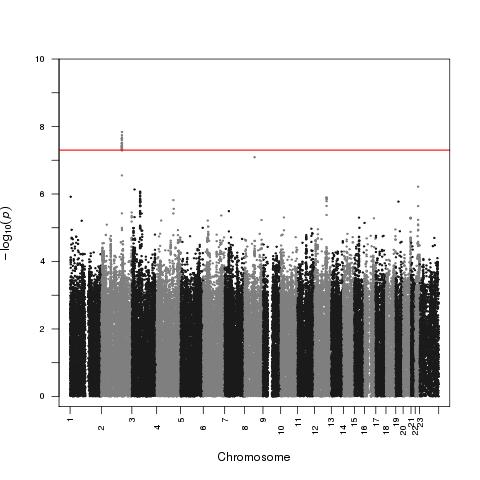


**Supplementary Figure 2.** Q-Q plot for the combined GWAS and COGS analyses for all breast cancer cases. The *y* axis represents the observed –log_10_ P value, and the *x* axis represents the expected –log_10_P value. The red line represents the expected distribution under the null hypothesis of no association.


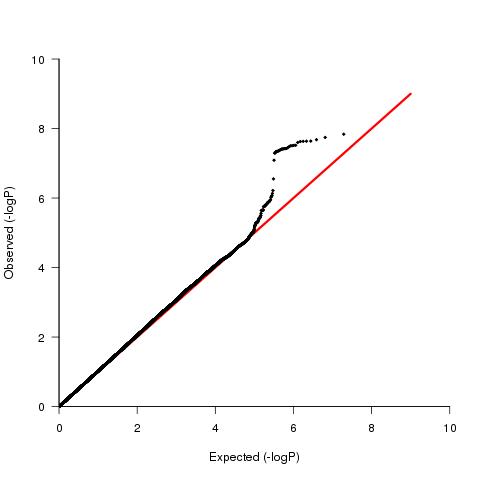


**Supplementary Figure 3.** Association plot for the final combined GWAS and COGS analyses for ER-positive cases. The P values of the association between each SNP and breast cancer survival were obtained by cox regression analyses with adjustment for principle components for each study and then combined. The *y* axis shows the –log_10_P values of each SNP analyzed, and the *x* axis shows their chromosome position. The red horizontal line represents P=5x10^-8^.


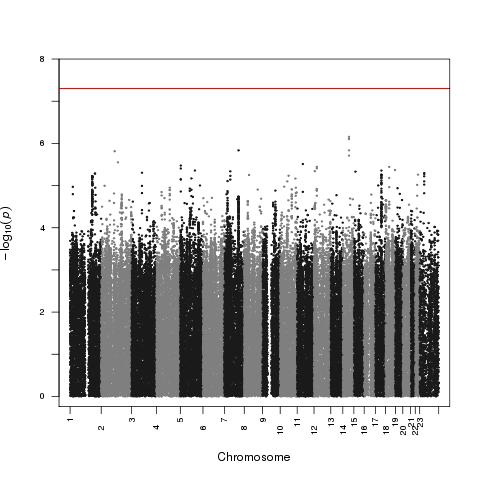


**Supplementary Figure 4.** Q-Q plot for the combined GWAS and COGS analyses for ER-positive cases. The *y* axis represents the observed –log_10_P value, and the *x* axis represents the expected –log_10_P value. The red line represents the expected distribution under the null hypothesis of no association.


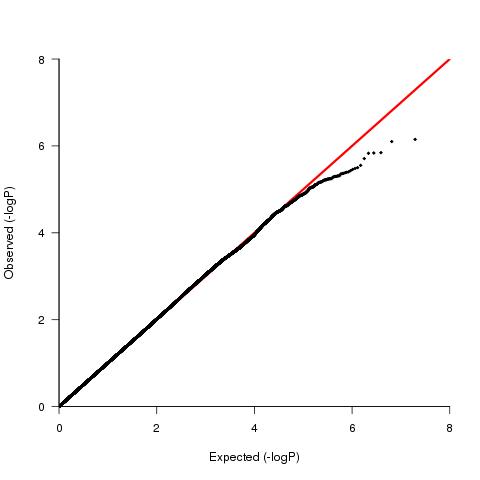


**Supplementary Figure 5.** Per-allele breast cancer-specific survival HR for rs148760487 on chromosome 2 by study based on imputed data with pre-phasing. The Hazard Ratio (HR) for each study is denoted by a black diamond. The 95% confidence interval (95%CI) is represented by a black line. Values outside of the range depicted are represented by an arrow. The P value for heterogeneity is shown using the I^2^ statistic.


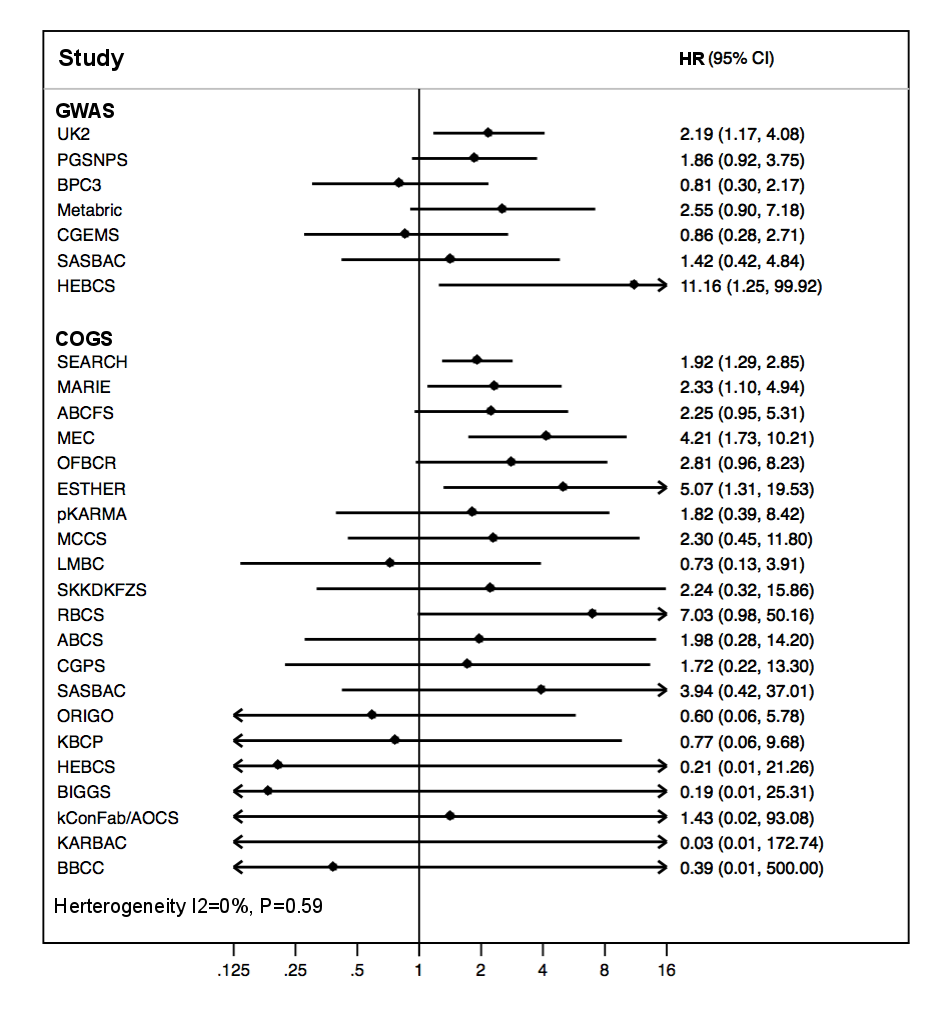


**Supplementary Figure 6.** Per-allele breast cancer-specific survival HR for rs2059614 on chromosome 11 by study for ER-negative breast cancer patients, based on imputed data with pre-phasing. The Hazard Ratio (HR) for each study is denoted by a black diamond. The 95% confidence interval (95%CI) is represented by a black line. Values outside of the range depicted are represented by an arrow. The P value for heterogeneity is shown using the I^2^ statistic..


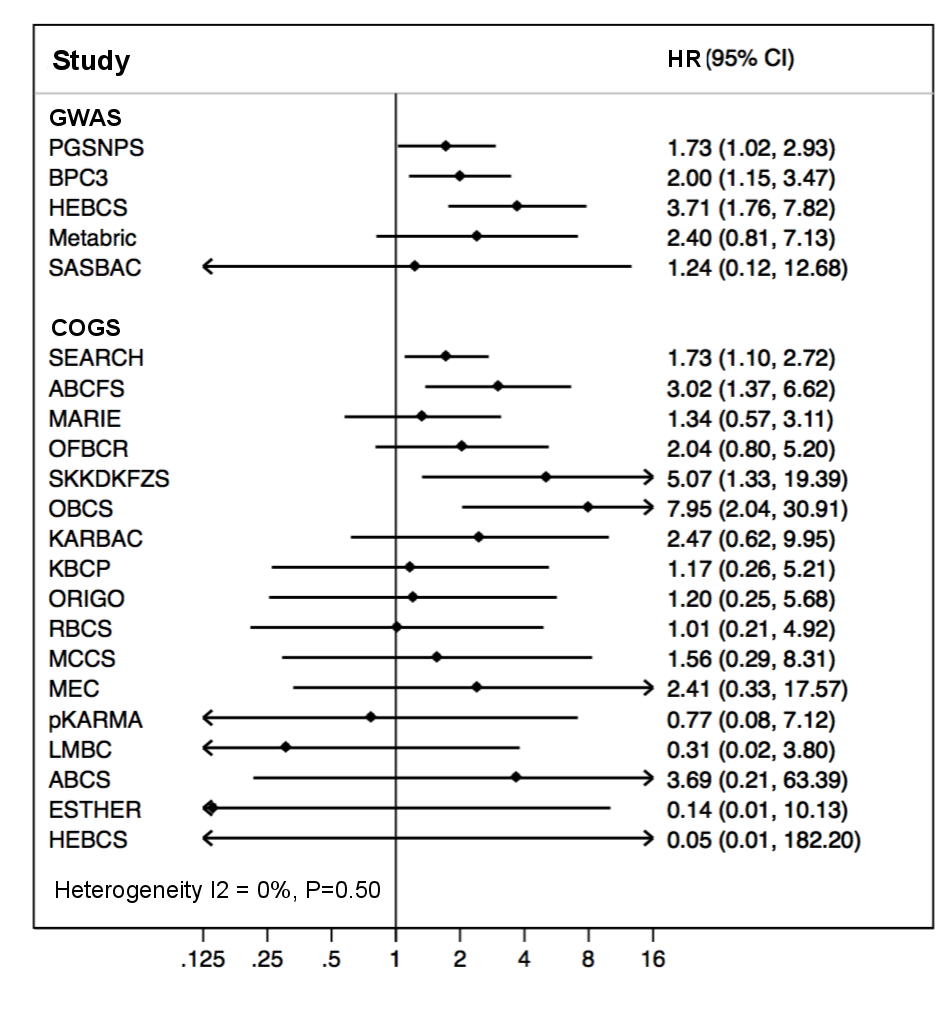


**Supplementary Figure 7.** Box plot of A) *EI24* and B) *CHEK1* expression by rs2059614 genotype in normal breast epithelium from the METABRIC study. The *x* axis represents the genotype of the SNP of interest. The *y* axis represents the gene expression values of the specified genes.


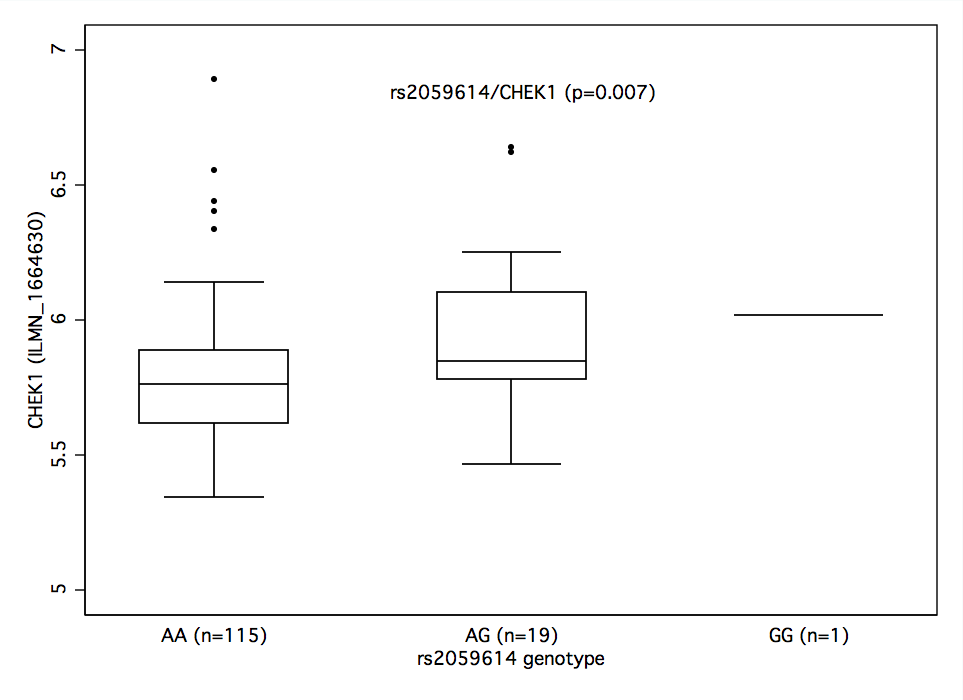

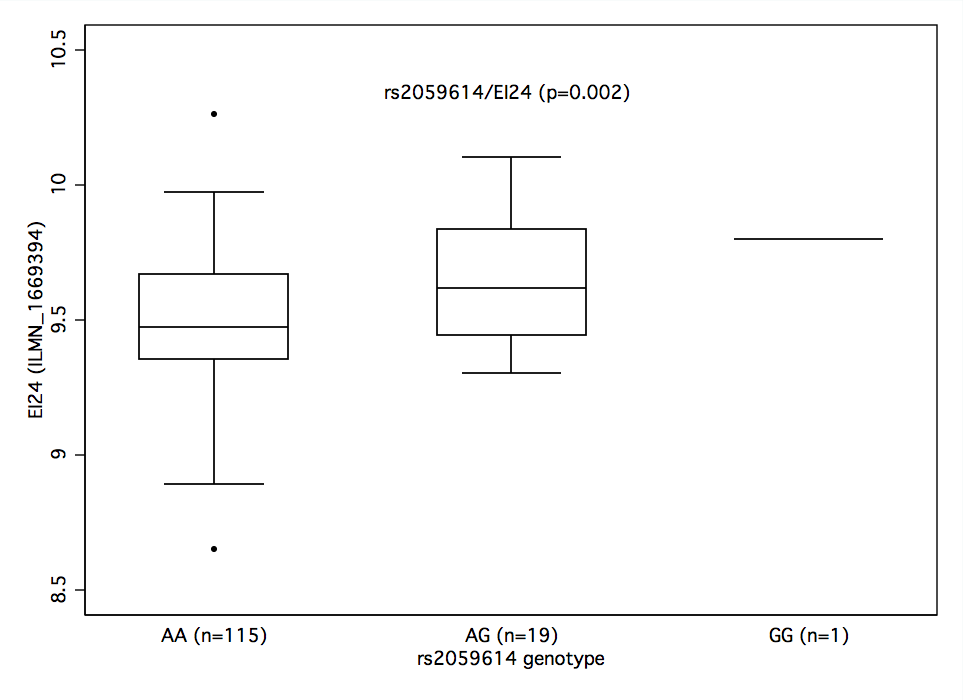


A

B

**Supplementary Figure 8.** Kaplan-Meier plot of effect of *CHEK1* and *EI24* expression on relapse-free survival of breast cancer patients using KM Plotter (**refer to Reference 34 in the main text)**: A) effect of *CHEK1* in ER-positive patients; B) effect of *CHEK1* in ER-negative patients; C) effect of *EI24* in ER- positive patients; D) effect of *EI24* in ER-negative patients. A two-sided log-rank test was used to calculate the P values.


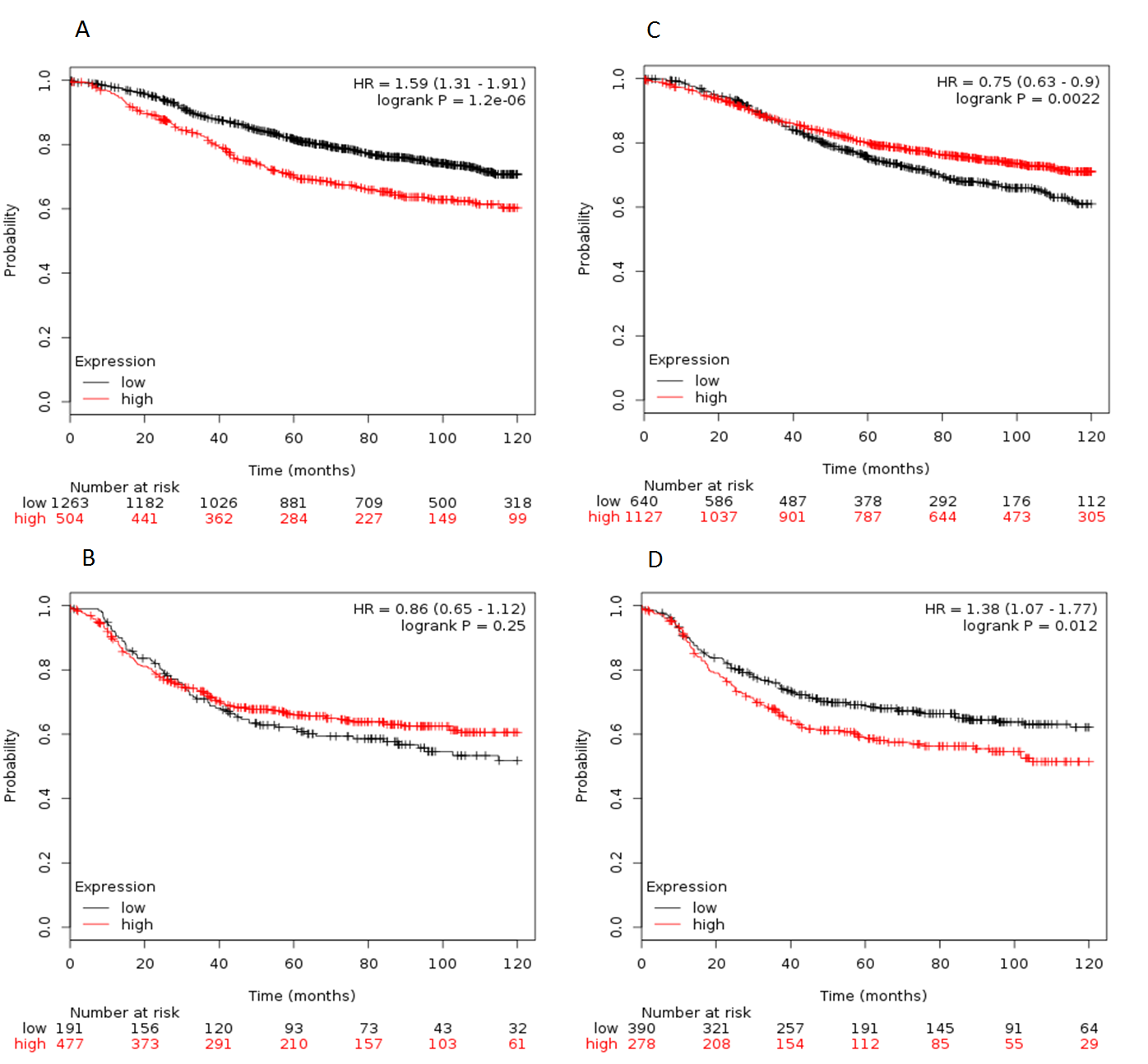


**Supplementary Figure 9.** Regional association plots displays the strength of genetic association (-log10 P) versus chromosomal position (MB), where each dot represents an imputed SNP. The purple horizontal line represents the threshold for genome-wide statistical significance (P = 5 × 10^-8^). Gene structures are depicted with the direction of transcription indicated by arrows. Enhancer locations, as defined in references (refer to References 41 and 42 in the main text),are shown where elements overlapping the best associated SNPs are labelled with their predicted target genes, *IFIH1* and *FAP* in (A). In (B), the blue stripe marks the position of rs2059614, and the red and green enhancers are predicted to target *EI24* and *CHEK1*, respectively. Positions of poorly imputed, common SNPs are shown.


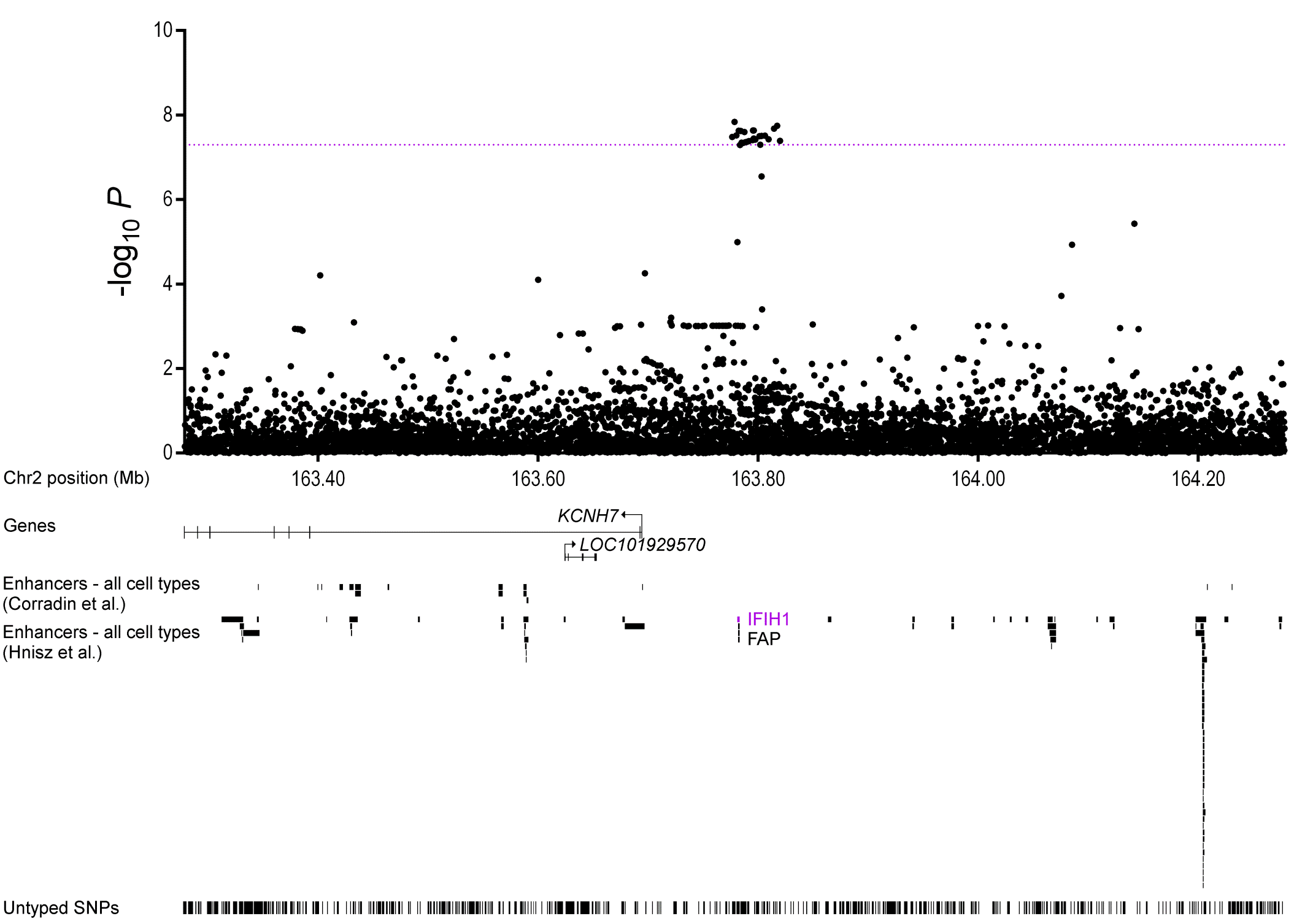

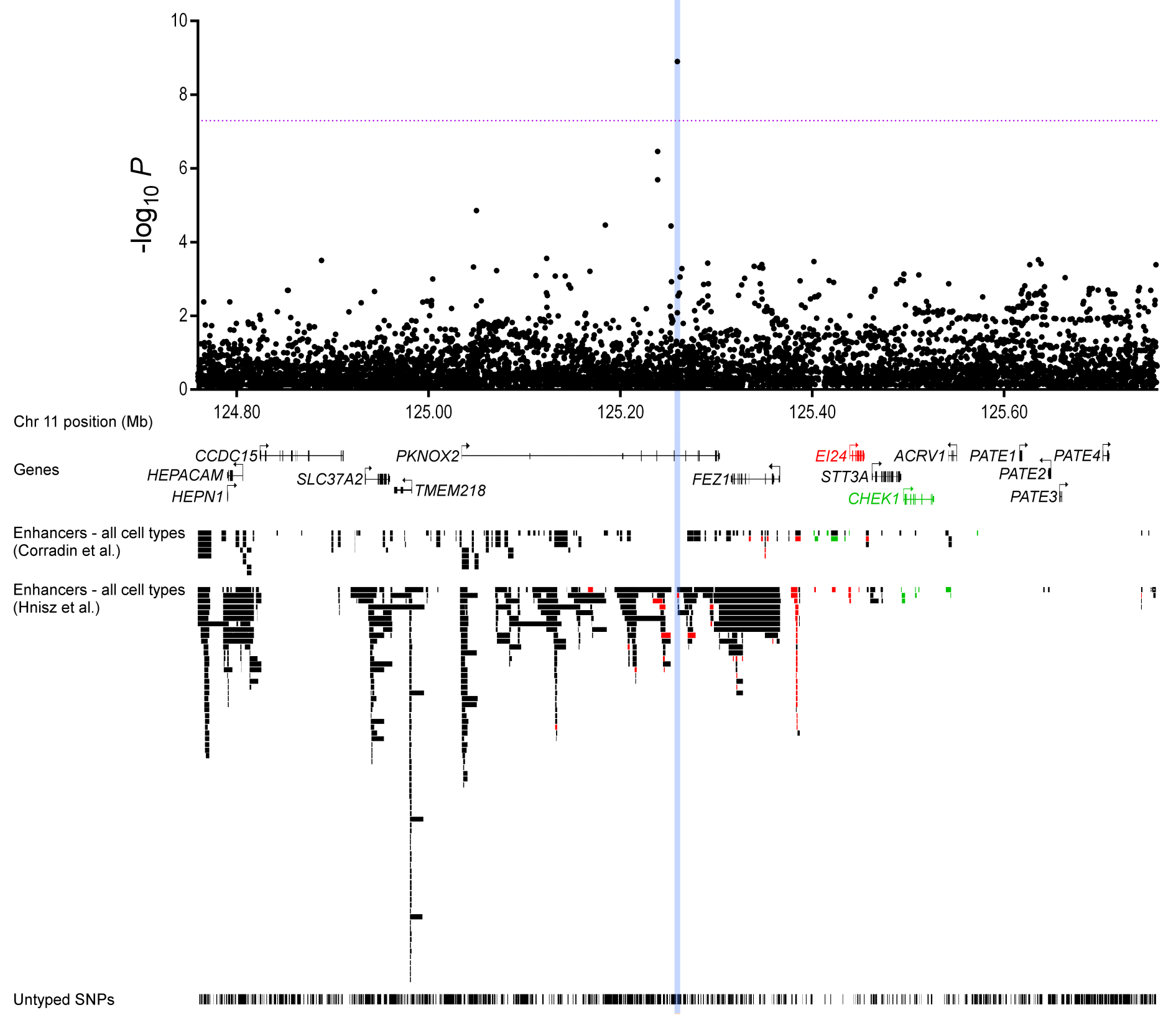


A

B

**Supplementary Figure 10.** Power plots for A) all breast cancers cases; B) ER-negative breast cancer cases. The *x* axis represents the Hazard Ratio and the *y* axis represents the power (*Beta*).The colors represent different Minor Allele Frequencies (MAF).


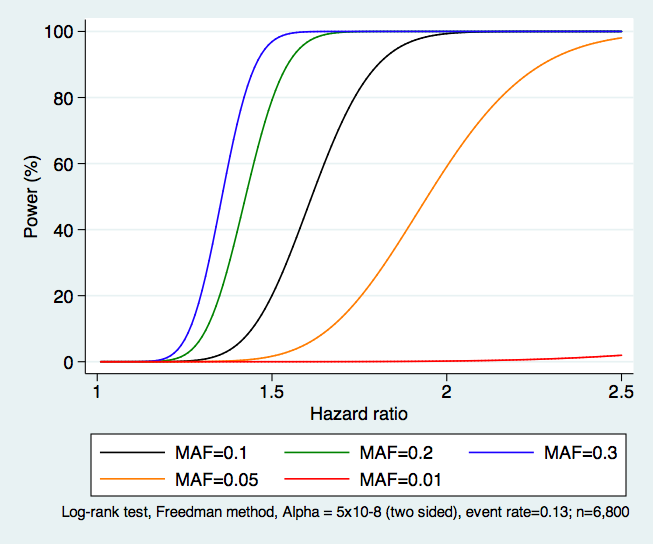

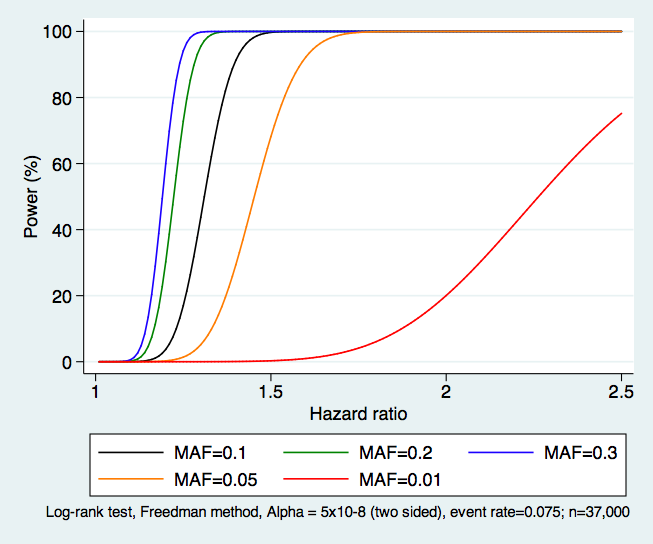


A

B
